# Australian pharmacists' experiences and perspectives in implementing a chronic kidney disease screening service in community pharmacies: A qualitative study

**DOI:** 10.1016/j.rcsop.2025.100643

**Published:** 2025-08-21

**Authors:** Ayana Korsa, Ines Krass, Connie Van, Wubshet Tesfaye, Natasa Gisev, Anh Tran, Rita McMorrow, Breonny Robson, Judith Fethney, Vincent Versace, Kamal Sud, Lukas Kairaitis, David Johnson, Judy Mullan, Sanjyot Vagholkar, Ronald L. Castelino

**Affiliations:** a Sydney Pharmacy School, Faculty of Medicine and Health, The University of Sydney, NSW, Australia; b School of Pharmacy, Institute of Health Sciences, Wallaga University, Nekemte, Ethiopia; c School of Pharmacy and Pharmaceutical Sciences, Faculty of Health, Medicine and Behavioural Sciences, The University of Queensland, QLD, Australia; d National Drug and Alcohol Research Centre, University of New South Wales, NSW, Australia; e NHMRC Clinical Trials Centre, The University of Sydney, NSW, Australia; f Department of General Practice and Primary Care, The University of Melbourne, VIC, Australia; g Kidney Health Australia, VIC, Australia; h Susan Wakil School of Nursing and Midwifery, Faculty of Medicine and Health, The University of Sydney, NSW, Australia; i Deakin Rural Health, School of Medicine, Faculty of Health, Deakin University, VIC, Australia; j Nepean Clinical School, Faculty of Medicine and Health, The University of Sydney, NSW, Australia; k Nepean Kidney Research Centre, Department of Renal Medicine, Nepean Hospital, NSW, Australia; l School of Medicine, Western Sydney University, NSW, Australia; m Department of Renal Medicine, Blacktown Mount Druitt Hospital, NSW, Australia; n Centre for Kidney Disease Research, The University of Queensland at Princess Alexandra Hospital, QLD, Australia; o Department of Kidney and Transplant Services, Princess Alexandra Hospital, QLD, Australia; p Graduate School of Medicine, Faculty of Science, Medicine and Health, University of Wollongong, NSW, Australia; q MQ Health General Practice, Macquarie University, NSW, Australia; r Department of Pharmacy, Blacktown Hospital, Sydney, NSW, Australia

**Keywords:** Chronic kidney disease screening, Community pharmacy services, Pharmacists' experience, Qualitative research

## Abstract

**Background:**

The emerging role of pharmacists in chronic kidney disease (CKD) care prompted the pharmacy-led screening and quality use of medicines in CKD trial (QUM-CKD), a pharmacy-led screening initiative to detect previously undiagnosed CKD and improve medication safety.Objective: To explore pharmacists' experiences and perspectives on the implementation of the QUM-CKD trial in Australian community pharmacies.

**Methods:**

A descriptive phenomenological qualitative approach was employed, involving in-depth, semi-structured telephone interviews with thirteen metropolitan and rural community pharmacists in the trial. Pharmacists were selected via purposive maximum variation sampling and were recruited mid-trial. Interviews were audio- recorded, transcribed verbatim, and thematically analysed using both deductive and inductive approaches in NVivo 14.

**Results:**

Most participating pharmacists reported having positive experiences with the trial's implementation. Facilitators of implementation included pharmacists' knowledge and beliefs, the availability of resources, support and training. The alignment with roles, values, and systems, along with perceived benefits of the service, the point-of-care testing service, a whole-team approach, and patient acceptance coupled with positive feedback, also facilitated implementation. Barriers included insufficient pharmacist staffing, time constraints, heavy workload, trial software and documentation issues, patients' lack of time, interest or unfavourable perceptions of the service, and interprofessional communication challenges between pharmacists and general practitioners (GPs). Pharmacists also suggested several potential improvements and expressed concerns about the sustainability of the service.

**Conclusions:**

Australian community pharmacists generally reported positive experiences in implementing the QUM-CKD trial. To ensure the service's success and sustainability, we recommend adequate pharmacy staffing, appropriate pharmacist remuneration, active stakeholder promotion and strong interprofessional collaboration. Pharmacists' suggestions for service improvement should also be considered.

## 1 Introduction

In recent decades, community pharmacy practice in high income countries including, the USA, UK, Canada and Australia has undergone a paradigm shift. While medicine dispensing remains a core responsibility, the profession is increasingly focused on delivering expanded healthcare services.^1^^,^^2^

Community pharmacists serve as highly accessible primary care providers who play key roles spanning from health education and promotion to the screening and management of both acute and chronic diseases.^2^, ^3^, ^4^ Community pharmacies provide reliable, accessible and convenient services to the public, supported by favourable locations and extended operating hours, making them an integral component of patient care pathways.^4^ With over 5900 community pharmacies handling more than 443 million patient visits annually—averaging at least 18 visits per person—and most Australians living within 2.5 km of a pharmacy, community pharmacies are among the most accessible healthcare settings in the country.^5^ In comparison, over 22 million Australians visited general practitioners (GPs) in 2023, averaging 7.6 services per person.^6^

Australian community pharmacists offer a range of government-funded services designed to prevent adverse drug events and enhance the quality use of medicines (QUM). These services include Home Medicines Review (HMR), Residential Medication Management Reviews (RMMR), MedsCheck, Diabetes MedsCheck, and support for drug dependence.^7^, 8., 9 Additionally, community pharmacists offer user-paid services such as vaccinations, health promotion and the screening and management of chronic conditions.^10^ A recent review in Australia found that pharmacist interventions have led to improved asthma control, enhanced detection of diabetes and cardiovascular risk factors, identification of drug-related problems, reduced smoking rates, and higher vaccination rates.^9^ Another study showed that over 95 % of consumers would support expanding pharmacy services and trust that pharmacists possess the necessary skills and knowledge to deliver them.^11^ This study also highlighted how these services have become a regular part of community pharmacy practice, suggesting that pharmacists have the knowledge, skills and motivation to expand their professional roles.^11^

Given pharmacists' proven success in chronic disease screening and management,^4^^,^^12^, ^13^, ^14^ community pharmacies are well-positioned to expand into screening for additional conditions such as chronic kidney disease (CKD)—a growing public health concern associated with serious health outcomes, including kidney failure and death. To address this growing concern, a pharmacy-based screening and quality use of medicines in CKD (QUM-CKD) trial was launched in Australia.^15^ This pioneering trial, funded by the Australian Government's Medical Research Future Fund 2020, is focused on CKD screening and the provision of QUM in CKD.^15^ In brief, QUM-CKD trial is a multi-centre, pragmatic, two-level cluster randomised controlled trial designed to assess the effectiveness of combining QKidney® risk assessment and point-of-care testing (POCT) with QUM services, compared to QKidney® risk assessment alone, in improving the detection rate of previously undiagnosed CKD and reducing the use of medications considered problematic in people with CKD.^15^

While evidence supports the benefits of pharmacy-based screening and other services for various diseases,^9^ the translation of this knowledge into practice and the adoption of expanded community pharmacy services in Australia have faced challenges.^4^^,^^16^^,^^17^ The gap between evidence and implementation may arise from the challenges and complexity involved in shifting from traditional pharmacy practice (e.g., medicines supply) to a health service-focused approach.^4^ To bridge this gap, it is crucial to understand the implementation process and identify the key factors that influence the successful adoption of these services.^18^ Doing so would provide a thorough understanding of the barriers to and enablers of implementation and the potential improvements and sustainability of the intervention. Furthermore, acquiring detailed insights into the experiences of community pharmacists in implementing new professional services could be key to ensuring the practicality and long-term success of these interventions.

Therefore, this study aimed to investigate the experiences and perspectives of community pharmacists involved in implementing the QUM-CKD trial and to explore their views on the service's sustainability and potential improvements if it were to be more broadly adopted in the future.

## 2 Methods

### 2.1 Study design

A descriptive phenomenological qualitative study was conducted using in-depth, semi-structured telephone interviews to gain insights into the lived experiences and perspectives of community pharmacists participating in the QUM-CKD trial. This qualitative approach was well-suited to the study's aim, which focused solely on pharmacists' description of their lived experiences and views regarding the implementation of the trial. A telephone interview was found suitable due to the geographical spread of the national trial and because some interviewees were unfamiliar with commonly used video conferencing platforms such as Zoom.

Initially, community pharmacies involved in the trial were selected using a purposive maximum variation sampling technique.^19^ Pharmacy champions—pharmacists nominated by their respective pharmacies to lead the trial's patient recruitment, screening and data collection—were then invited to participate in interviews, forming the study sample. This sampling method included a diverse group of trial participants, enabling the collection of a broad spectrum of pharmacists' views and/or experiences in implementing the trial. The study was reported following the consolidated criteria for reporting qualitative research (COREQ), a widely used 32-item checklist for reporting qualitative studies.^20^

### 2.2 Study setting

Community pharmacies participating in the trial were located in four Australian states: Australian Capital Territory (ACT), New South Wales (NSW), Queensland (QLD) and Victoria (VIC). These pharmacies were situated in both metropolitan and rural areas. Pharmacy champions from the selected pharmacies, representing the four states and various geographical locations, were invited to participate in the interview.

Pharmacists were interviewed via telephone during working hours, with interviews scheduled at a pre-arranged date and time between September and November 2023. Interviews were conducted at the midpoint of the trial (3 months) to gain insights into factors influencing the trial's implementation and to address any issues prior to its completion. One experienced qualitative researcher (CV) conducted all interviews.

### 2.3 Participants

A purposive sample of 13 pharmacists, each representing one pharmacy participating in the CKD trial, was selected to take part in the mid-trial interview. Pharmacies were first selected based on representation of both metropolitan and rural locations to broadly reflect the distribution of the Australian population, following this dichotomy (71 % of the Australian population resides in Metropolitan areas, i.e., those classified as Modified Monash 1).^21^ The definition of metropolitan and rural applied in this study was policy-relevant and consistent with that of the Australian Government Department of Health and Aged Care's definition used in the Rural Health Multidisciplinary Training Program.^21^

Participating pharmacies were then stratified into two groups based on their ability to meet the trial recruitment target of 30 participants over 6 months, which equated to 1.25 participants per week.^15^ Pharmacies that met or exceeded this target by the time of interview were classified as high-recruiting (HR) pharmacies, while those below the target were classified as low-recruiting (LR) pharmacies. The project manager (WT) then generated two lists of pharmacies—high and low recruiting—drawn from the entire pool of trial pharmacies in both metropolitan and rural areas. These pharmacies were further stratified by trial arm (Intervention versus Control).^15^ Ultimately, 13 pharmacists (one pharmacy champion from each selected pharmacy), representing a mix of recruitment performance, geographical remoteness and trial arms, were contacted for phone interviews. Reimbursement was provided to participating pharmacies (but not directly to individual pharmacists) for patient recruitment, screening and referral to GPs. However, no incentives were offered to compensate pharmacists for the time spent participating in interviews.

### 2.4 Data collection

The interview guide was initially developed by the research team (WT and RLC) based on a review of the available literature and was then refined by another team member (IK) utilising relevant constructs from the Consolidated Framework for Implementation Research (CFIR).^22^ Finally, the guide was discussed and revised by the research team to ensure its relevance and clarity. CFIR is a comprehensive determinant framework designed to provide a detailed understanding of the factors influencing the successful implementation of innovations in health services research. It consists of five domains: innovation (the intervention being implemented), outer setting (the external system where the innovation is introduced, such as the primary care), inner setting (the characteristics of the organisation where the innovation is applied, for example, a community pharmacy), individuals (the roles and attributes of those involved in the implementation), and the implementation process (the activities and strategies used to implement the intervention).^22^ This framework was chosen because it has been used to investigate the experiences and viewpoints of pharmacists in implementing new professional pharmacy services.^16^^,^^23^

The interview questions explored the participating pharmacists' overall experience with the QUM-CKD trial, their perceptions of the service's design and implementation, their views on how the service was operationalised, and their experiences with the recruitment process. They were also asked about their thoughts on the training and support provided by research staff, the acceptance of the service by the community or consumers, and the impact of the trial on their professional services, pharmacy businesses, and interprofessional collaboration. Finally, pharmacists were invited to offer suggestions for improving and sustaining the service if it were to be implemented in the future. Follow-up questions and prompts were also employed to facilitate more in-depth discussions (Table S1).

The interviews were audio-recorded and stored on the University of Sydney's cloud server, with access restricted to the research team for confidentiality. Similarly, a custom-designed software was used to collect quantitative data for the trial. This database was accessible only to the research team and the pharmacists in the database-opted pharmacies.

All interviews were stored in a folder, with each pharmacy assigned an identifier along with its recruitment performance (e.g., PharmId 1721 HR). One researcher (AK) and an independent transcription service provider transcribed the recordings verbatim into Microsoft Word. The transcriptions were then cross-checked against the audio files to ensure accuracy and completeness. Data collection continued until information saturation was reached, meaning no new themes or ideas emerged.

### 2.5 Data management and analysis

The full transcripts were imported into NVivo software for Windows (Lumivero: version 14, released in 2023) for further analysis. A descriptive thematic analysis was employed to identify key themes and sub-themes. An initial codebook was developed based on Weir et al.'s^24^ CFIR-based systematic review of 39 studies, which identified three overarching themes and fourteen constructs that influenced the national implementation of innovations in community pharmacies. These themes included pharmacy staff engagement, operationalisation of the innovation, and external engagement.^24^ AK thoroughly reviewed each transcript line-by-line to become familiar with the data and prepared the codebook that was later revised by two other researchers (IK and CV). A blend of deductive and inductive coding^25^ was applied to identify key concepts and to organise the data into themes and sub-themes. Responses that aligned with the initial codes were initially coded deductively, while unique responses and additional themes (e.g. sustainability, future improvements) were coded inductively.^26^ A deductive approach guided the identification of CFIR-aligned concepts, supplemented by inductive coding to capture emergent themes relevant to the research questions. Finally, three researchers (AK, IK, and CV) compared the emergent themes and sub-themes, resolving any disagreements through discussion until consensus was achieved.

## 3 Results

Thirteen in-depth, semi-structured interviews were conducted between September and November 2023. All interviews took place over the telephone and lasted between 20 and 37 min, with an average duration of about 26 min. Most participants (69.2 %) were from metropolitan areas, and more than half (53.8 %) were from high-recruiting pharmacies (Table 1).

**Table 1:** Characteristics of community pharmacies (*n* = 13).

Variables	N (%)
Trial arm	
Intervention group	8 (61.5)
Control group	5 (38.5)
State	
ACT	2 (15.4)
NSW	7 (53.8)
QLD	3 (23.1)
VIC	1 (7.7)
Region	
Metropolitan	9 (69.2)
Rural	4 (30.8)
Pharmacy recruitment performance	
High recruiters	7 (53.8)
Low recruiters	6 (46.2)
Recording duration (minutes), mean (range)	26 (20–37)

ACT, Australian Capital Territory; NSW, New South Wales; QLD, Queensland; VIC, Victoria.

Several themes and subthemes, aligned with the CFIR and the thematic framework developed by Weir et al.,^24^ were identified. Table 2 summarised the themes and subthemes that emerged from the pharmacists' interviews in the QUM-CKD trial, with additional quotes included as supplementary data in Table S2.

**Table 2:** Themes and sub-themes emerging from pharmacists' interviews.

Themes	Sub-themes
Overall pharmacist experience	Positive experience
	Negative experience
Pharmacy staff engagement	Knowledge and beliefs about the intervention
	Compatibility with roles or values
	Pharmacy staffing
	Relative advantage
	Strategies used to improve participation
Operationalisation of the innovation	Availability of resources
	Access to knowledge and information
	Design quality and packaging
	Compatibility with systems
	Complexity
External engagement	Pharmacist-GP interactions
	Impact on professional collaboration
	Patient perspectives
Future improvements and sustainability	Suggestions to improve patient participation
	Suggestions to improve the database and documentation
	Pharmacist perspectives on sustainability
	Charging clients for the service

### 3.1 Overall pharmacist experience

Most pharmacist participants reported positive experiences with implementing the screening service. They highlighted high patient participation, increased awareness of CKD risk factors for patients, and enhanced patient engagement.

*We had a positive experience. A lot of the patients that I have spoken to were excited and very willing to do it (PharmId 1721 HR).*

*I would say [the screening service] is efficient… raising [awareness of] the risk of having medications… the consequence of the side effects that might happen with these medications. It is good to spotlight this area… [CKD screening] was and is still needed (PharmId 1713 HR).*

However, a few participants expressed dissatisfaction due to their inability to use the software program and failure to meet recruitment targets.

*The biggest challenge for me is with the computer, the software, the program. I struggle to use the program (PharmId 1714 LR).*

*Overall experience, I think I was very excited initially, but when we started promoting it, we could not get the numbers we aimed for (PharmId 1738 LR).*

### 3.2 Factors influencing implementation

#### 3.2.1 Pharmacy staff engagement

##### 3.2.1.1 Knowledge and beliefs about the intervention

The study participants expressed diverse opinions about the service. Most participants from high-recruiting pharmacies believed that the screening service would play a significant role in maintaining patients' kidney health and recommended CKD screening for everyone, especially those with risk factors for CKD. They also underscored that pharmacies should offer similar services for other health conditions.

*I think [the screening service] will have a big impact. I think [kidney health] is an important thing to keep an eye on, considering the statistics on CKD… asymptomatic and stuff like that. I think it is important for everybody, especially diabetics, to get screened (PharmId 1721 HR).*

In contrast, a participant from a low-recruiting pharmacy felt that offering the service in pharmacies was inappropriate, as doctors are already monitoring kidney function in medical centres.

*No [service not suitable to run in pharmacy] … doctors are already monitoring [kidney function] (PharmId 1748 LR).*

##### 3.2.1.2 Compatibility with roles or values

Most pharmacists in high-recruiting pharmacies believed the service was beneficial to patients and aligned with their goals and plans. They emphasised the importance of providing more professional services, rather than relying exclusively on conventional ones like dispensing or compounding medications in community pharmacies.

*I found it very helpful for patients and something that should be part of pharmacy. We should look at renal function, and [creatinine] clearance, and check for drug doses. So, it goes hand in hand with what we want to do here (PharmId 1762 HR).*

##### 3.2.1.3 Pharmacy staffing

The most frequently mentioned barrier by participants, regardless of pharmacy recruitment performance level, was the lack of sufficient pharmacy personnel to conduct the screening service. Respondents identified staff workload and lack of time as challenges to service implementation, highlighting the need for additional staff and funding to run the service effectively.

*Our major holdup is we do not have the staff–the extra staff–to grab those patients while they are waiting. I suppose you need the funding to support an extra staff member to be able to fulfill the service (PharmId 1747 LR).*

*It is not as easy as we wish it to be … like 45* min *or one hour with the patient away from our daily duties. That is the thing we need to consider (PharmId 1713 HR).*

Despite the challenges, some pharmacies implemented strategies to tackle the issue, such as scheduling patients when multiple pharmacists were on duty and involving pharmacy assistants and front-of-shop staff.

##### 3.2.1.4 Relative advantage

Many participants from both high- and low-recruiting pharmacies indicated that the service would benefit patients, the pharmacy and pharmacists, fostering greater customer interaction. Additionally, they noted that the trial improved their professional roles, enhanced workforce capability (or encouraged them to think outside the box), and strengthened their engagement and relationships with patients.

*I think [the service] adds value to the patients and indirectly to the pharmacy and the pharmacists. We prefer to have a good service like that here in the pharmacy (PharmId 1713 HR).*

*This is a strong therapeutic relationship we can build with the patients… they come asking different questions in many ways, and they rely on us (PharmId 1743 HR).*

##### 3.2.1.5 Strategies used to improve participation

In accordance with the trial protocol,^15^ participating pharmacies appointed a pharmacy champion, while all other pharmacy staff members (pharmacists, interns, pharmacy assistants, and technicians) were encouraged to assist with recruitment. As a result, pharmacists in both high- and low-recruiting pharmacies recognised the involvement of the pharmacy team in the recruitment process as a key factor in facilitating the implementation of the service. In high-recruiting pharmacies, involving non-pharmacist staff, such as pharmacy assistants and front-of-shop staff, in the recruitment process led to increased patient participation rates.

*We have got our main shop assistant starting the conversation with people all the time, and sort of screening … then it is me, my intern, and the other pharmacist, who has probably done most (PharmId 1746 HR).*

In low-recruiting pharmacies, pharmacy champions had trained and engaged their pharmacy assistants, however, patient participation rates were low due to factors including patients' lack of time and/or interest.

*The staff… they know how to recruit customers. They check everyone with the prescription coming… give them the leaflet and ask them if they want to participate (PharmId 1714 LR).*

*Time is another [barrier]. Some of [the patients] did not want to [participate] as well (PharmId 1738 LR).*

Other strategies were also employed to enhance trial participation. For example, one interviewee from a high-recruiting pharmacy noted that briefing potential participants on the trial's key information resulted in higher recruitment rates compared to allowing them to read the information at their convenience.

*To go out and talk to people and give them a brief explanation of what it is about, and the information that they can read themselves. We found that works well… (PharmId 1721 HR).*

Another pharmacist suggested establishing a strong rapport with patients before requesting their participation.

*I would say just build up trust with the patients so they can trust you and listen to you… give them time, space, and information to think about, they can get back to you (PharmId 1738 LR).*

#### 3.2.2 Operationalisation of the innovation

##### 3.2.2.1 Availability of resources

Prior to the trial's implementation, promotional materials, such as eligibility cards, flyers, posters and flow charts, were provided to each participating pharmacy. Additionally, each pharmacy was required to adhere to the standard operating procedures (SOPs) and complete the pharmacy operations manual workbook.^15^ When asked about the usefulness of these resources, participants from both high- and low-recruiting pharmacies confirmed that the materials were valuable in planning and executing the screening service.

*The operations manual workbook was helpful… it was useful at the beginning (PharmId 1713 HR).*

*For me, it was the paperwork we received that was good… the resources that we have for patients, and the prior knowledge… that helped promote and do the trial (PharmId 1738 LR).*

Moreover, participants in both HR and LR groups valued the support provided by the research team, which greatly facilitated the process during the initial planning and execution of the trial.

*Having somebody come into the store and take us through how to do it one-on-one was great. When you have somebody come in, show you the paperwork, and explain how to do it in person, that makes it a lot easier (PharmId 1746 HR).*

##### 3.2.2.2 Access to knowledge and information

An online training course, accredited by the Pharmaceutical Society of Australia (PSA) and tailored to the control and intervention pharmacy groups, was developed and delivered through Medcast before the trial began.^15^ Most pharmacists in both high- and low-recruiting pharmacies found these modules valuable and informative, helping them better understand the trial process while also enhancing their knowledge, skills, and confidence in implementing the intervention. Additionally, some pharmacists emphasised the importance of hands-on training with the research staff, especially in learning how to operate the point-of-care testing (POCT) machine.

*When I completed the module, I was quite confident about going ahead and doing things (PharmId 1743 HR).*

*We did all those training things… that was a good way to be up-to-date on medications and health and how to use the program and everything (PharmId 1716 LR).*

*When VV [research staff] came up, we ran through [the POCT] in person, which made it a lot clearer (PharmId 1747 LR).*

##### 3.2.2.3 Design quality and packaging

Most pharmacists in both high- and low-recruiting groups reported that the POCT was well-received and that patients found the simple finger prick followed by delivery of results with minimal wait time, impressive.

*Patients were impressed… if you go to a clinical lab, the results come back after maybe 24* *h at least… having it in 10* *s is quite good (PharmId 1713 HR).*

However, some pharmacists from low-recruiting pharmacies expressed dissatisfaction with the trial software program, noting its slow performance and the lack of prompts to indicate missing information or guide them on what to do when errors occur.

*Easy to collect information but the problem is when we put the data into the computer. We try to go through to the next step but cannot … we do not know what is missing (PharmId 1714 LR).*

*One or two screenings were not made because the database was slow, and we did not know how to give them a number [risk assessment or QKidney® score] (PharmId 1738 LR).*

##### 3.2.2.4 Compatibility with systems

Several pharmacists from high-recruiting pharmacies noted that the screening service was compatible with their current working systems and easy to implement in their settings.

*We like to be doing professional services. So, this just goes with what our culture is, our business is. It just closes in with like part of what we do (PharmId 1764 HR).*

##### 3.2.2.5 Complexity

Pharmacists from both HR and LR groups reported challenges in implementing the trial due to a lack of GP follow-ups. They explained that either patients did not visit their GPs, or GPs were reluctant to conduct kidney health tests and provide feedback to the pharmacies for completing the follow-ups.

*We have not had many people come back to us for a follow-up… the doctors have told us that [our patients] have not been in to see them straight away. It is difficult to get a follow-up and fill that part out (PharmId 1721 HR).*

Age restrictions (patients being ineligible due to age) and delays in screening caused by paperwork were other operational challenges reported by pharmacists in both high- and low-recruiting groups, which affected their recruitment targets.

*Sometimes [people co*min*g to us] did not fit the [inclusion] criteria. I had a lot of young people who were quite interested, but they were not eligible. I had lost a few because of the age restriction (PharmId 1716 LR).*

*We had to run the trial for longer than expected… because we had to print… it [occupied us] for almost 45* *min to an hour [per] patient. That limited the number of recruitments, unfortunately (PharmId 1713 HR).*

#### 3.2.3 External engagement

##### 3.2.3.1 Patient perspectives

In general, pharmacists from both HR and LR groups received positive feedback from patients involved in the trial. For most pharmacists from high-recruiting pharmacies, the key factors behind patient acceptance of the service were the pharmacies' accessibility and quick service delivery.

*I would say [the patients] appreciate it. Most patients feel [the service] adds value, especially since it is a very quick service. The community appreciates it (PharmId 1713 HR).*

*[Patients] told us that it is important to do this sort of stuff in the pharmacy because we are easy to access (PharmId 1721 HR).*

Likewise, a pharmacist from a low-recruiting pharmacy felt that the service was well-received by patients as it helped them gain a better understanding of their kidney health.

*[The patients] were very happy with how [the service] was offered to them and [how they got] a better, clearer understanding of what their kidney health was (PharmId 1716 LR).*

However, some patients also expressed negative perceptions, either because they did not recognise the pharmacist-delivered screening service or because they regularly visited their GPs and were not interested in taking the test with pharmacists.

*[Patients] do not recognise that pharmacists can do a screening like this. So, whenever we start to talk about kidneys, they are like … my doctor is taking care of that, my doctor knows about it (PharmId 1738 LR).*

*Only the ones who do not see the doctor are interested… the ones who see the doctor regularly do not need to check [their renal function] again (PharmId 1714 LR).*

Additionally, pharmacists from both high- and low-recruiting pharmacies often cited patients' lack of time, interest or reluctance to participate as the main barriers to implementation.

*Time, time. Patient lacking time is your biggest barrier (PharmId 1746 HR).*

*They are not interested in it (PharmId 1721 HR).*

##### 3.2.3.2 Pharmacist-GP interactions

The level of interaction between pharmacists and GPs varied between high-recruiting and low-recruiting pharmacies. Some pharmacists from high-recruiting pharmacies reported that doctors referred eligible participants to the pharmacy for the screening service and conducted follow-up tests when required.

*We just have one GP surgery across the road that we talk to regularly. I think they referred one or two people to come in and have a chat with me. I have heard back from the GP. They have done follow-up tests (PharmId 1721 HR).*

In contrast, pharmacists from low-recruiting pharmacies did not reach out to GPs or patients for follow-up tests and had no interaction with the participating GPs at all.

*No, I have not needed to call a GP yet. I have not had a GP call about it… the patients have not told me if they have [seen their GP] (PharmId 1716 LR).*

##### 3.2.3.3 Impact on professional collaboration

Regarding interprofessional collaboration, pharmacists from both high- and low-recruiting pharmacies stated that the trial had minimal to no impact on their partnership with GPs.

*I would not say [the screening service] added any value at all to the GPs, knowing that we are running it in the pharmacy here (PharmId 1713 HR).*

*Even if [the patients] see the GP [and] give the referral letter to the GP … the GPs do not order the test (PharmId 1714 LR).*

#### 3.2.4 Future improvements and sustainability

##### 3.2.4.1 Suggestions to improve patient participation

Pharmacists offered several suggestions to improve patient recruitment and participation. They highlighted the importance of making consent forms and similar documents accessible online, allowing patients to sign them from their mobile phones or iPads easily. They also recommended providing refresher training for pharmacy assistants and students to better prepare them for supporting the recruitment process.

*The consent forms and stuff… it might be easier to use [an] online consent form as well for people. Something that you can put on an iPad or on their phone that they can find (PharmId 1721 HR).*

*Maybe a little bit of training that aimed at some of the pharmacy assistants and the students. Like a little module, for example, [like the] one we did for COVID-19 vaccination training (PharmId 1746 HR).*

Some participants also felt that the trial was not adequately promoted to key stakeholders, including GPs and pharmacists. They suggested that better promotion of the service before the trial's implementation would have greatly enhanced patient participation and encouraged interprofessional collaboration.

*It was not well promoted to the GPs. If it was well promoted to the GPs and the pharmacist, I would say that could have had a huge impact… even GPs would send patients (PharmId 1713 HR).*

##### 3.2.4.2 Suggestions to improve the database and documentation

Regarding documentation, some pharmacists from high-recruiting pharmacies recommend simplifying the process. For example, they suggested sending health-related quality-of-life questionnaires to patients who had agreed to participate in the trial, instead of having pharmacists complete them immediately during the screening at the pharmacy.

*I think we would make it easier if we had less documentation to fill out, instead of having multiple consent forms and multiple pages to fill in… sending a questionnaire would be easier in the future (PharmId 1713 HR).*

Similarly, most pharmacists from both high- and low-recruiting pharmacies encountered issues with the trial software. They believed that if the program was improved and operated more smoothly, it would enhance their ability to implement the trial more effectively.

*We had a problem with the software initially… [even] now I am still not happy with the software. I wish it could be better and run better (PharmId 1725 HR).*

*The software is slow. If that can be fixed, we can just do it (PharmId 1738 LR).*

##### 3.2.4.3 Pharmacist perspectives on sustainability

Most pharmacists from high-recruiting pharmacies believed that the service was effective enough to be widely implemented in community pharmacies in the future, potentially making a significant impact on maintaining kidney health.

*It is a very good service to apply in more community pharmacies in the future (PharmId 1712 HR).*

*[If implemented widely, the service] would have a huge impact. I think it is so important that this is part of what pharmacy does. We were very happy that this POCT was available. We were not aware [it existed] (PharmId 1762 HR).*

On the other hand, while pharmacists believed that offering professional services was more rewarding for both themselves and the pharmacy business compared to traditional services, they observed that these services did not have an immediate impact on their pharmacy income, as they did not attract new customers.

*I think it has been good to offer another service. That is the way pharmacy businesses must go. Professionally, I think something like that is far more rewarding than just the basic dispensing (PharmId 1746 HR).*

*It is the same [had no impact on our pharmacy business] because we only do it for our regular [patients], we have not attracted any extra customers at all (PharmId 1725 HR).*

##### 3.2.4.4 Charging clients for the service

Finally, when asked about patients' willingness to pay for the screening service in the future, some pharmacists noted that patients were currently willing to participate because the service was free. They suggested that patients might be reluctant to pay for it as a standalone service unless it included additional assessments and counselling. They also pointed out that patients with access to bulk-billing medical centres tend to avoid out-of-pocket costs, which could affect the feasibility of charging for the service in the future.

*I do not think they would pay for the screening. If that screening involved kind of more counselling and assessment, perhaps they would pay. But again, in my demographic area, being next to a bulk-billing medical centre, they do not like to pay (PharmId 1746 HR).*

## 4 Discussion

Community pharmacy services are rapidly evolving to offer a wider range of health services focused on improving patient health outcomes. To adapt effectively to this shift, various service models have been trialed and successfully implemented in community pharmacies worldwide.^4^^,^^13^^,^^14^^,^^16^^,^^26^^,^^27^ In this context, the present study, embedded in the QUM-CKD trial, is the first to examine the experiences and perspectives of pharmacists in implementing novel CKD screening services in Australian community pharmacies. It provides valuable evidence and contributes to the existing literature on the key factors that drive the successful implementation of novel pharmacy services.

Generally, community pharmacists reported high patient engagement with the CKD screening service, increased awareness of CKD risk factors for patients, and a positive contribution to maintaining patients' kidney health. Several factors enabled successful implementation including the pharmacists' beliefs in the service and its alignment with their roles, values or systems. In addition, the availability of resources, support from research staff and training, the involvement of the entire pharmacy team, and positive patient perceptions and feedback were key to effective implementation. Barriers to service implementation included pharmacy staffing issues, software and documentation issues, minimal GP follow-ups and pharmacist-GP interactions, as well as patient-related factors such as lack of time, interest or recognition of pharmacists' services. These identified enablers of, and barriers to implementation align closely with the framework proposed by Weir et al.^24^ Further, participating pharmacists posited several practical strategies to improve patient participation (such as promoting the service to stakeholders, training non-pharmacist staff to assist with recruitment), and streamlining the technological integration and documentation processes. They also shared their perspectives on the sustainability of the service for broader implementation.

Participating pharmacists reported that patients were generally satisfied with the service, particularly appreciating the quick turnaround of POCT results compared to clinical laboratories. Similar findings have been reported in previous studies,^13^^,^^26^ which noted that the use of POCT devices was crucial in supporting the implementation. Additionally, most participating pharmacists reported that the service received significant patient support, acceptance and positive feedback. This was particularly attributed to the service being free and the convenient accessibility of pharmacies, with similar results reported elsewhere.^13^^,^^16^^,^^26^ These findings are supported by the evidence that whilst patients' attitudes and perceptions toward an innovation largely impact its implementation,^28^ the convenience and availability of free services are among the crucial factors that motivate most patients to engage in novel community pharmacy services.^13^^,^^26^^,^^27^

Most pharmacists believed that the CKD screening service was an important additional screening program that community pharmacies could offer, helping to alleviate the burden of CKD due to its high prevalence and often asymptomatic nature. Studies have shown that pharmacists are keen to expand their roles to include providing professional services^26^^,^^29^ and strongly believe that offering screening services would benefit the health of their patients.^16^^,^^26^ Further, pharmacists' perceptions of innovation play a crucial role in influencing implementation. If they do not recognise the value of a service, they may face challenges in adopting it.^23^ This was highlighted by a pharmacist from a low-recruiting pharmacy, who believed the screening service was unnecessary in the pharmacy, as GPs were already offering it at medical centres. Accordingly, having positive beliefs and thoughts toward service is pivotal for its successful implementation.^28^ However, individual motivation must be augmented by systemic support, and other factors influencing behaviour, such as personal abilities and organisational culture, need to be addressed for widespread success in transforming pharmacy practice.^16^^,^^29^

The perceived relative advantage of the service by pharmacists was another key enabling factor identified, consistent with the findings of previous studies.^24^^,^^26^^,^^30^ This highlights that when pharmacists perceive a clear relative advantage of a service—both for their patients and for their own professional practice—they are more likely to embrace, sustain and actively promote its implementation.

Another key factor facilitating the trial's implementation was its alignment with the pharmacists' roles, values and systems. Most pharmacists from high-recruiting pharmacies viewed the service as integral to their goals and aspirations, finding it compatible with their existing workflow. This accords with the findings of Weir et al.^24^ and Shoemaker et al.^30^ However, it contrasts with the findings of Alzubaidi et al.,^26^ who reported that pharmacists in the United Arab Emirates (UAE) struggled to integrate the screening service into their workflow. This difficulty may be attributed to restrictive pharmaceutical legislation in the UAE, which limited the services pharmacists could offer due to concerns about hazard management and the potential for misuse in community pharmacies.^26^ In contrast, community pharmacies in Australia are typically encouraged to provide professional services, including disease screening.^8^ Furthermore, some pharmacists in the current study engaged their non-pharmacist staff and used interpersonal strategies, such as building rapport with potential participants, to enhance recruitment. This suggests that future interventions should adopt a whole-of-pharmacy approach, where all staff are informed, involved and equipped to support implementation efforts. These findings align with those of other studies which underscore the use of a team-based approach and the adoption of various strategies to enhance recruitment.^4^^,^^13^^,^^16^ They also highlight the importance of involving pharmacy technicians or assistants^4^^,^^28^^,^^31^ for the successful implementation of new pharmacy services.

Study resources—such as the operations manual, advertising materials and flowcharts—were frequently cited as key enablers of service operationalisation, particularly for planning, raising awareness and engaging participants. Pharmacists also highlighted the value of strong research team support, including timely responses, site visits, and online pre-trial training, as critical to effective implementation. These findings underscore the importance of accessible resources and proactive support in facilitating implementation, consistent with previous studies that identify structured materials, training, and research staff involvement as essential to successful implementation.^4^^,^^13^^,^^30^

The trial protocol^15^ outlined specific eligibility criteria (ages 35 to 74), which some pharmacists indicated impacted recruitment. It also required GPs to follow up with referred patients and send the results to the pharmacies. However, several pharmacists stated that limited GP follow-up hindered the completion of referral outcomes for patients. Despite this, most participating pharmacists found the operationalisation of the service to be relatively straightforward. Furthermore, consistent with the findings of others,^4^^,^^13^^,^^16^^,^^18^^,^^24^^,^^26^^,^^30^^,^^31^ inadequate pharmacy staff, time constraints and heavy workloads were major implementation barriers. In this context, one study highlighted that increased staff capacity positively impacted the adoption of change, while insufficient staff had a negative effect.^23^ Additionally, a study examining the determinants of implementing Australian government-funded community pharmacy services identified pharmacists' lack of time as a major barrier to implementation.^18^ Nevertheless, in our study, some pharmacies sought to address these challenges by scheduling patients when more than one pharmacist was on duty and by involving pharmacy assistants and front-of-shop staff.

Another key barrier to implementation included patient-related factors, such as lack of time, interest or reluctance, and unawareness of pharmacist services, all of which have been previously documented.^4^^,^^16^^,^^18^^,^^26^ Patients were found to have a limited understanding of extended pharmacy services^16^^,^^18^ and their lack of interest often challenged recruitment and retention in the trial.^4^ In our study, some patients may have been too busy to participate, as the screening service could take up to 45 min, while most typically visit pharmacies for quicker services, such as purchasing medications or filling prescriptions. Therefore, raising awareness or informing the public about the service and its benefits could be key to its successful implementation.

The final frequently reported barrier was the perceived negative attitudes of GPs toward the service, along with limited communication and collaboration with pharmacy staff. Despite the trial protocol^15^ outlining a clear strategy for patient referrals including a software-generated letter detailing patient risk status, POCT results and QUM issues, with copies sent to the patients' nominated GPs, pharmacists from both high- and low-recruiting pharmacies consistently reported limited interactions with GPs, low levels of collaboration, and in several instances, no responses from GPs.

These findings suggest that limited collaboration and communication between pharmacists and GPs may significantly hinder the successful implementation of pharmacy-led interventions. Similar issues have been reported in other studies,^3^^,^^16^^,^^24^^,^^26^ highlighting barriers related to interprofessional communication and collaboration within the healthcare system. To help mitigate this barrier, early engagement strategies^13^ and efforts to foster positive collaborative experiences^32^ have been recommended.

For future improvements, pharmacists proposed strategies like enhancing service promotion, training pharmacy assistants to support recruitment, and optimising software and documentation. For sustainability, they recommended that community pharmacies better focus on offering professional services such as CKD screening, which they viewed as more professionally rewarding than routine practices like dispensing. They also believed that the service would have a significant impact if adopted more broadly. However, pharmacists felt that patients were drawn to the service primarily because it was free and that they would be reluctant to pay for it unless it included additional assessments and counselling. This aligns with a previous finding where pharmacists believed that charging patients fees would deter service uptake.^13^ In contrast, other Australian studies revealed that most patients highly valued community pharmacy services such as asthma management^33^ and CKD risk assessment^34^ and were willing to pay for them. Therefore, further research is needed to explore how perceived value influences patients' willingness to adopt and pay for new pharmacy services.

## 5 Strengths and limitations

The main strength of this study lies in its sampling approach, which included representations from both metropolitan and rural pharmacies, thereby broadly reflecting the distribution of the Australian population. Additionally, purposive maximum variation sampling facilitated a deeper exploration of the differences between high- and low-recruiting pharmacies in implementing the trial. Another notable strength is the use of the theoretical framework developed by Weir et al.,^24^ which identifies the key determinants for implementing innovation in community pharmacies, grounded in the CFIR,^22^ a widely recognised framework in implementation science. The data aligned well with this framework, enabling the examination of the factors that contribute to the successful implementation of CKD screening services in Australian community pharmacies. The reliability, accuracy, and trustworthiness of the findings were ensured through a detailed interview guide, thorough thematic analysis, and independent cross-checking of codes by three researchers. However, it is also important to acknowledge that the study may have been influenced by recall bias due to the extended time gap between the start of the trial and when the interviews were undertaken. Furthermore, incorporating the perspectives of other stakeholders, such as patients and GPs, would provide a more comprehensive understanding of the implementation process within the broader primary care system.

## 6 Conclusions

The study found that Australian community pharmacists generally had positive experiences implementing the CKD screening service, supporting its potential for broader national adoption. It identified key facilitators and barriers to successful implementation and highlighted variations across pharmacies, which may be attributed to factors such as staff attitudes, perceived service value, motivation and communication or problem-solving strategies. The findings also emphasised the dynamic interplay between pharmacists, patients and GPs in shaping the successful implementation of pharmacy-led services, highlighting the need to strengthen interprofessional collaboration. Additionally, pharmacists' perspectives on improving and sustaining the service offer valuable direction for future research and implementation efforts.

The following are the supplementary data related to this article.

Supplementary material All data from the study are presented in the main text, with additional data available in a supplementary file on Research in Social and Administrative Pharmacy online. Table S1. Pharmacist interview guide.
Table S2. Themes, sub-themes, and illustrative quotes. Supplementary material

## Funding

This project was supported by the Medical Research Future Fund of the Australian Government's Primary Healthcare Initiative (MRFQI00008). The funding body has no role in the study design, data collection, analysis, interpretation or manuscript preparation.

## Availability of data and materials

Transcripts from individual pharmacist participants are not accessible to the public.

## Ethics approval

This study underwent ethical review and received approval from the Human Research Ethics Committee of the University of Sydney (protocol no. 2022/044). The pharmacists participating in the QUM-CKD project had signed informed consent forms before their involvement and were informed that mid-trial and exit interviews would be conducted. Nevertheless, before starting the interviews, pharmacists were reminded that participation was voluntary and their responses would be kept confidential and anonymous.

## Consent for publication

Participants consented to be audio recorded, understanding that their results would be de-identified before publication.

## CRediT authorship contribution statement

**Ayana Korsa:** Writing – review & editing, Writing – original draft, Methodology, Investigation, Formal analysis, Data curation. **Ines Krass:** Writing – review & editing, Supervision, Methodology, Investigation, Formal analysis, Conceptualization. **Connie Van:** Writing – review & editing, Methodology, Investigation, Formal analysis, Data curation. **Wubshet Tesfaye:** Writing – review & editing, Project administration, Methodology, Investigation, Conceptualization. **Natasa Gisev:** Writing – review & editing, Investigation. **Anh Tran:** Writing – review & editing, Investigation. **Rita McMorrow:** Writing – review & editing, Investigation. **Breonny Robson:** Writing – review & editing. **Judith Fethney:** Writing – review & editing. **Vincent Versace:** Writing – review & editing. **Kamal Sud:** Writing – review & editing, Investigation. **Lukas Kairaitis:** Writing – review & editing, Investigation. **David Johnson:** Writing – review & editing, Investigation. **Judy Mullan:** Writing – review & editing, Investigation. **Sanjyot Vagholkar:** Writing – review & editing, Investigation. **Ronald L. Castelino:** Writing – review & editing, Supervision, Resources, Project administration, Methodology, Investigation, Funding acquisition, Conceptualization.

## Declaration of competing interest

The authors declare that they have no known competing financial interests or personal relationships that could have appeared to influence the work reported in this paper.
