# Supplementary material for: Australian pharmacists' experiences and perspectives in implementing a chronic kidney disease screening service in community pharmacies: A qualitative study
Source: Explor Res Clin Soc Pharm. 2025 Aug 21;20:100643. doi: 10.1016/j.rcsop.2025.100643 (PMC12408408; doi:10.1016/j.rcsop.2025.100643)
Supplement: Supplementary material — All data from the study are presented in the main text, with additional data available in a supplementary file on Research in Social and Administrative Pharmacy online. Table S1. Pharmacist interview guide. Table S2. Themes, sub-themes, and illustrative quotes. [file mmc1.docx]

Table S1. Pharmacist interview guide.

Preamble

Good morning/afternoon. May I speak to …………………. (pharmacist)

*My name is …………………. and I am from the University of Sydney. We are interested in conducting a brief interview with you (or a group of pharmacists) to determine your experiences in the* ***Pharmacy-led Screening and Quality Use of Medicines in Kidney Disease Trial,*** *a study* *funded by the Government under the Medical Research Future Fund scheme as outlined in the participant information sheet you received at the beginning of the trial. The interview will take approximately 15-30 minutes, and we are flexible to schedule it at your convenience. We would appreciate your willingness to participate. It's important to note that your participation is entirely voluntary, and any information you provide will be kept confidential and anonymised.*

| **Interview guide** |
| --- |
| **Pharmacist experience:**  *What has been your overall experience?*  Prompts   - What went well? - Who was involved in the service? Promoting, Recruiting, and Service Delivery. - How many pharmacists, including interns, are involved? - Do you feel it was adequately staffed? - Who decided to sign up your pharmacy for the trial? - How many pharmacists were eligible to do the screening – and were involved in the screening? - What could be improved? - How did the process operate in your pharmacy? - Counselling room? - Technology requirement? (e.g., Computer or tablet) - Disruption to workflow or efficiencies of pharmacy? - What factors enabled the process to be integrated? - Resources involved in terms of time/staff. - What aspects of the study procedures represented a change to your usual way of practice/workload (and was this change positive or negative)? - (Group 1) What aspects of the intervention were most challenging to implement?   *How well did the online training equip you to deliver the service?*  Prompts   - The online modules are provided through Medcast. Do you recall? - What were some positive aspects of the training modules? - What were some negative aspects of the training modules? - Knowledge – kidney disease risk factors, the role of screening, Qkidney® tool, and (Group 1 – quality use of medicines in kidney disease) - Confidence - Skills – (patient education) - Motivation - Recruitment - What could be improved? - For Group 1 – what was your experience of the POCT device training? And in terms of POCT use for kidney function screening in pharmacies, what went well? What could have been improved?   *How well did the implementation/operation manual support in delivering the service?*  Prompts   - What aspects were most helpful? - What could be improved? - What advice or strategies would you recommend to other pharmacists to improve rates of recruitment or quality of service delivery? - What other resources did you have to help deliver the service (human resources, computer)?   *What was it like to recruit participants for this study?*  Prompts   - Easy or difficult - What strategies did you employ to recruit participants? - Were you able to enrol the targeted number of study participants? What prevented you from achieving your target? - Could you give me examples of reasons participants provided for declining to participate in this study? - Usefulness of promotional materials - Overall, would you consider the risk assessment service a success?   *What was your experience of the data collection and documentation?*  Prompts   - What went well with the study database employed? - What could be improved? - Easy to set up and use?   *What did you think of the service protocol?*  Prompts   - What worked well/not so well? - Any suggestions for improvement? - What elements of support from the research team were helpful?   *How does this screening role fit with your role as a community pharmacist?*  Prompts   - In what ways does it extend or challenge your scope of practice? - How does it influence your identity as a pharmacist? (or does it distract from more relevant duties) - Does the service fit with the business model of your pharmacy?   *Did the use of POCT improve the quality use of medicines service you provided?*  Prompts   - How does this service fit into other QUM interventions in your pharmacy if any, e.g., MedsCheck/Diabetes MedsCheck? |
| **Consumer perspective:**  *How was the screening service received by your clients/local community?*  Prompts   - What did your patients think about the screening? - Which aspects of the service were most/least needed by clients? - What aspects of the service were most/least appreciated by clients? - (Group 1) How did consumers respond to getting follow-up after screening? - What aspects of screening helped to persuade consumers to take up a referral to their GP? - How do you think this screening service has impacted your clients' view on services (such as screening) within a pharmacy? - Have you received any other feedback from participants? |
| **GP/Specialist interaction:**  *How did you interact with GPs during the program?*  Prompts   - Personally contacted GPs. - Used the General Practitioner Information Letter to contact GPs.   - - All local GPs     - Selected GPs     - Other   *How have GPs been responding to your referrals? What happened after the referral?*  Prompts   - Personal (telephone/face-to-face) communication - Written communication between   *What was the overall impact on your professional relationship with local GPs?* |
| **Other impacts:**  *What other impacts did you experience because of offering the screening service?*  Prompts   - Business. - Demands for other services. - Invitation to participate in other professional activities e.g., seminars, committees, etc. - *What impact has participation in this service had on your attitudes to providing services generally – pharmacist and other staff?* - *How well do you think this will influence your future pharmacy practice?* |
| **The Future:**  *How much demand do you think there is for a service like this in community pharmacies?*  Prompts   - Do you believe that there is perceived value from the patient’s perspective?   *Would you like to continue delivering this service and if so, how?*  Prompts   - The service as it is or in a modified form. - Remuneration – was the reimbursement enough, too low, or too high? - Resourcing – tools, staff, facilities, - Promotion.   Assuming funding on a similar level, how well would the profession implement the service? (Quality, recruitment numbers)  Assuming funding on a similar level, would the same service levels be sustainable over time by the profession?  Finally, if there was anything that you could change or do differently when implementing a similar screening service in your pharmacy in the future, what would it be? |

***Thank you for your time and assistance in completing this survey.***

*This study is being conducted by the Sydney Pharmacy School, at the University of Sydney and funded by the Medical Research Future Fund*

Table S2. Themes, sub-themes, and illustrative quotes.

| Major domains | Themes | Sub-themes | Examples |
| --- | --- | --- | --- |
| Overall experience | Pharmacist experience | Positive experience | *We had a positive experience. A lot of the patients that I have spoken to were excited and very willing to do it (PharmId 1721 HR).*  *I would say [the screening service] is efficient… raising [awareness of] the risk of having medications… the consequence of the side effects that might happen with these medications. It is good to spotlight this area… [CKD screening] was and is still needed (PharmId 1713 HR).* |
|  |  | Negative experience | *The biggest challenge for me is with the computer, the software, the program. I struggle to use the program (PharmId 1714 LR).*  *Overall experience, I think I was very excited initially, but when we started promoting it, we could not get the numbers we aimed for (PharmId 1738 LR).* |
| Factors influencing implementation | Pharmacy staff engagement | Knowledge and beliefs about the intervention | *I think [the screening service] will have a big impact. I think [kidney health] is an important thing to keep an eye on, considering the statistics on CKD… asymptomatic and stuff like that. I think it is important for everybody, especially diabetics, to get screened… (PharmId 1721 HR).*  *No [service not suitable to run in pharmacy] … doctors are already monitoring [renal function] (PharmId 1748 LR).* |
|  |  | Compatibility with roles or values | *I found it very helpful for patients and something that should be part of pharmacy. We should look at kidney function, and [creatinine] clearance, and check for drug doses. So, it goes hand in hand with what we want to do here (PharmId 1762 HR).* |
|  |  | Pharmacy staffing | *Our major holdup is we just do not have the staff – the extra staff – to grab those patients while they are waiting. I suppose you need the funding to support an extra staff member to be able to fulfill the service (PharmId 1747 LR).*  *It is not as easy as we wish it to be … like 45 minutes or one hour with the patient away from our daily duties. That is the thing we need to consider (PharmId 1713 HR).* |
|  |  | Relative advantage | *I think [the service] adds value for the patients and indirectly to the pharmacy and the pharmacists. We prefer to have a good service like that here in the pharmacy (PharmId 1713 HR).*  *This is a strong therapeutic relationship we can build with the patients… they come asking different questions in many ways, and they rely on us (PharmId 1743 HR).* |
|  |  | Strategies used to improve participation | *We have got our main shop assistant starting the conversation with people all the time, and sort of screening ... then it is me, my intern, and the other pharmacist, who has probably done most (PharmId 1746 HR).*  *The staff… they know how to recruit customers. They check everyone with the prescription coming… give them the leaflet and ask them if they want to participate (PharmId 1714 LR).*  *To go out and talk to people and give them a brief explanation of what it is about, and the information that they can read themselves. We found that works well… (PharmId 1721 HR).*  *I would say just build up trust with the patients so they can trust you and listen to you… give them time, space, and information to think about, they can get back to you (PharmId 1738 LR).* |
|  | Operationalisation of the innovation | Availability of resources | *The operations manual workbook was helpful… it was useful at the beginning (PharmId 1713 HR).*  *For me, it was the paperwork we received that was good… the resources that we have for patients, and the prior knowledge… that helped promote and do the trial (PharmId 1738 LR).*  *Having somebody come into the store and take us through how to do it one-on-one was great. When you have somebody come in, show you the paperwork, and explain how to do it in person, that makes it a lot easier (PharmId 1746 HR).* |
|  |  | Access to knowledge and information | *When I completed the module, I was quite confident about going ahead and doing things (PharmId 1743 HR).*  *We did all those training things… that was a good way to be up to date on medications and health and how to use the program and everything (PharmId 1716 LR).*  *When VV [research staff] came up, we ran through [the POCT] in person, which made it a lot clearer… (PharmId 1747 LR).* |
|  |  | Design quality and packaging | *Patients were impressed… if you go to a clinical lab, the results come back after maybe 24 hours at least… having it in 10 seconds is quite good (PharmId 1713 HR).*  *I think [the service design] is okay. I think it is like other pharmacy services – if we promote, yes, we can do it (PharmId 1738 LR).*  *Easy to collect information, but the problem is when we put the data into the computer. We try to go through to the next step, but cannot … we do not know what is missing (PharmId 1714 LR).*  *One or two screenings were not made because the database was slow, and we did not know how to give them a number [risk assessment or QKidney® score] (PharmId 1738 LR).* |
|  |  | Compatibility with systems | *We like to be doing professional services. So, this just goes with what our culture is, our business is. It just closes in with like part of what we do (PharmId 1764 HR).* |
|  |  | Complexity | *We have not had many people come back to us for a follow-up… the doctors have told us that [our patients] have not been in to see them straight away. It is difficult to get a follow-up and fill that part out (PharmId 1721 HR).*  *Sometimes [people coming to us] did not fit the [inclusion] criteria. I had a lot of young people who were quite interested, but they were not eligible. I had lost a few because of the age restriction (PharmId 1716 LR).*  *We had to run the trial for longer than expected… because we had to print… it [occupied us] for almost 45 minutes to an hour [per] patient. That limited the number of recruitments, unfortunately (PharmId 1713 HR).* |
|  | External engagement | Pharmacist-GP interactions | *We just [have] one GP surgery across the road that we talk to regularly. I think they referred one or two people to come in and have a chat with me. I have heard back from the GP. They have done follow-up tests (PharmId 1721 HR).*  *No, I have not needed to call a GP yet. I have not had a GP call about it… the patients have not told me if they have [seen their GP] (PharmId 1716 LR).* |
|  |  | Impact on professional collaboration | *I would not say [the screening service] added any value at all to the GPs, knowing that we are running it in the pharmacy here (PharmId 1713 HR).*  *Even if [the patients] see the GP [and] give the referral letter to the GP … the GPs do not order the test (PharmId 1714 LR).* |
|  |  | Patient perspectives | *I would say [the patients] appreciate it. Most patients feel [the service] adds value, especially since it is a very quick service. The community appreciates it (PharmId 1713 HR).*  *[Patients] told us that it is important to do this sort of stuff in the pharmacy because we are easy to access (PharmId 1721 HR).*  *[The patients] were very happy with how [the service] was offered to them and [how they got] a better, clearer understanding of what their kidney health was (PharmId 1716 LR).*  *[Patients] do not recognise that pharmacists can do a screening like this. So, whenever we start to talk about kidneys, they are like … my GP is taking care of that, my GP knows about it (PharmId 1738 LR).*  *Only the ones who do not see the GP are interested… the ones who see the GP regularly do not need to check [their renal function] again (PharmId 1714 LR).*  *Time, time. Patient lacking time is your biggest barrier (PharmId 1746 HR).*  *They are not interested in it (PharmId 1721 HR).* |
| Improvement and sustainability | Future Improvements | Suggestions to improve patient participation | *The consent forms and stuff… it might be easier to use [an] online consent form as well for people. Something that you can put on an iPad or on their phone that they can find (PharmId 1721 HR).*  *It was not well promoted to the GPs. If it was well promoted to the GPs and the pharmacist, I would say that could have had a huge impact… even GPs would send patients (PharmId 1713 HR).*  *Maybe a little bit of training that aimed at some of the pharmacy assistants and the students. Like a little module, for example, [like the] one we did for COVID-19 vaccination training (PharmId 1746 HR).* |
|  |  | Suggestions to improve database and documentation | *I think we would make it easier if we had less documentation to fill out instead of having multiple consent forms and multiple pages to fill in… sending a questionnaire would be easier in the future (PharmId 1713 HR).*  *We had a problem with the trial software initially… [even] now I am still not happy with the software. I wish it could be better and run better (PharmId 1725 HR).*  *The software is slow. If that can be fixed, we can just do it (PharmId 1738 LR).* |
|  | Sustainability | Charging clients for the service | *I do not think they would pay for the screening. If that screening involved kind of more counselling and assessment, perhaps they would pay. But again, in my demographic area, being next to a bulk-billing medical centre, they do not like to pay (PharmId 1746 HR).* |
|  |  | Pharmacist perspectives on sustainability | *It is a very good service to apply in more community pharmacies in the future (PharmId 1712 HR).*  *[If implemented widely, the service] would have a huge impact. I think it is so important that this is part of what pharmacy does. We were very happy that this POCT was available. We were not aware [it existed] (PharmId 1762 HR).*  *I think it has been good to offer another service. That is the way pharmacy businesses must go. Professionally, I think something like that is far more rewarding than just the basic dispensing (PharmId 1746 HR).*  *It is the same [had no impact on our pharmacy business] because we only do it for our regular [patients], we have not attracted any extra customers at all (PharmId 1725 HR).* |
